# Research Priorities in Limb and Task-Specific Dystonias

**DOI:** 10.3389/fneur.2017.00170

**Published:** 2017-05-03

**Authors:** Sarah Pirio Richardson, Eckart Altenmüller, Katharine Alter, Ron L. Alterman, Robert Chen, Steven Frucht, Shinichi Furuya, Joseph Jankovic, H. A. Jinnah, Teresa J. Kimberley, Codrin Lungu, Joel S. Perlmutter, Cecília N. Prudente, Mark Hallett

**Affiliations:** ^1^Department of Neurology, University of New Mexico Health Sciences Center, Albuquerque, NM, USA; ^2^Institute for Music Physiology and Musicians’ Medicine (IMMM), Hannover University of Music, Drama and Media, Hannover, Germany; ^3^Functional and Applied Biomechanics Section, Rehabilitation Medicine, National Institute of Child Health and Development, National Institutes of Health, Bethesda, MD, USA; ^4^Division of Neurosurgery, Beth Israel Deaconess Medical Center, Boston, MA, USA; ^5^Division of Neurology, Department of Medicine (Neurology), Krembil Research Institute, University of Toronto, Toronto, ON, Canada; ^6^Robert and John M. Bendheim Parkinson and Movement Disorders Center, Mount Sinai Hospital, New York, NY, USA; ^7^Musical Skill and Injury Center (MuSIC), Sophia University, Tokyo, Japan; ^8^Department of Neurology, Baylor College of Medicine, Houston, TX, USA; ^9^Department of Neurology, Emory University School of Medicine, Atlanta, GA, USA; ^10^Department of Human Genetics, Emory University School of Medicine, Atlanta, GA, USA; ^11^Department of Pediatrics, Emory University School of Medicine, Atlanta, GA, USA; ^12^Department of Rehabilitation Medicine, Division of Physical Therapy and Rehabilitation Science, University of Minnesota, Minneapolis, MN, USA; ^13^Division of Clinical Research, National Institute of Neurological Disorders and Stroke, National Institutes of Health, Bethesda, MD, USA; ^14^Department of Neurology, Washington University School of Medicine, St. Louis, MO, USA; ^15^Department of Radiology, Washington University School of Medicine, St. Louis, MO, USA; ^16^Department of Neurosciences, Washington University School of Medicine, St. Louis, MO, USA; ^17^Department of Physical Therapy, Washington University School of Medicine, St. Louis, MO, USA; ^18^Department of Occupational Therapy, Washington University School of Medicine, St. Louis, MO, USA; ^19^Human Motor Control Section, National Institute of Neurological Disorders and Stroke, National Institutes of Health, Bethesda, MD, USA

**Keywords:** dystonia, limb, task-specific, research priorities, inhibition, deep brain stimulation, botulinum toxin

## Abstract

Dystonia, which causes intermittent or sustained abnormal postures and movements, can present in a focal or a generalized manner. In the limbs, focal dystonia can occur in either the upper or lower limbs and may be task-specific causing abnormal motor performance for only a specific task, such as in writer’s cramp, runner’s dystonia, or musician’s dystonia. Focal limb dystonia can be non-task-specific and may, in some circumstances, be associated with parkinsonian disorders. The true prevalence of focal limb dystonia is not known and is likely currently underestimated, leaving a knowledge gap and an opportunity for future research. The pathophysiology of focal limb dystonia shares some commonalities with other dystonias with a loss of inhibition in the central nervous system and a loss of the normal regulation of plasticity, called homeostatic plasticity. Functional imaging studies revealed abnormalities in several anatomical networks that involve the cortex, basal ganglia, and cerebellum. Further studies should focus on distinguishing cause from effect in both physiology and imaging studies to permit focus on most relevant biological correlates of dystonia. There is no specific therapy for the treatment of limb dystonia given the variability in presentation, but off-label botulinum toxin therapy is often applied to focal limb and task-specific dystonia. Various rehabilitation techniques have been applied and rehabilitation interventions may improve outcomes, but small sample size and lack of direct comparisons between methods to evaluate comparative efficacy limit conclusions. Finally, non-invasive and invasive therapeutic modalities have been explored in small studies with design limitations that do not yet clearly provide direction for larger clinical trials that could support new clinical therapies. Given these gaps in our clinical, pathophysiologic, and therapeutic knowledge, we have identified priorities for future research including: the development of diagnostic criteria for limb dystonia, more precise phenotypic characterization and innovative clinical trial design that considers clinical heterogeneity, and limited available number of participants.

## Introduction

The dystonias are a group of disorders characterized by sustained or intermittent muscle contractions causing abnormal and often repetitive movements, postures, or both (1, 2). Clinically the dystonias are classified according to the area of the body that is affected, their age at onset, temporal characteristics such as manner of onset or task specificity, and whether they are combined with other neurological or medical features. Etiologically, they are classified according to whether or not there is any associated brain pathology or evidence for a genetic basis. In this review, we will focus mainly on isolated limb dystonia that usually presents in adult life, most commonly is of unknown origin, and can be task-specific. We will summarize the current knowledge in the areas of clinical features, pathophysiology, as well as current therapeutic strategies. Then, we will identify priorities for future research based on the knowledge gaps revealed.

## Clinical Features

### Dystonia of the Upper Limb

In epidemiological studies conducted in different parts of the world, the most commonly affected regions of the body include the neck and craniofacial areas (3, 4). The upper limbs are the third most commonly affected area, with estimated crude prevalence rates of approximately 5–70 cases per million (3, 4). The majority of upper limb dystonias first emerge in adulthood, with approximately 10–20% progressing to other body regions over 5–10 years (5–7). Upper limb dystonias less commonly emerge in children; although when they do present in children, there is greater risk of progression to generalized dystonia (8). The commonly reported features of upper limb dystonia include abnormal extension or flexion of the wrist or fingers, pain in the hand or forearm, and tremulous movements. Sometimes, there is pain in the hand or forearm, but this is typically not a prominent symptom and may be due to excessive muscular contraction.

In writer’s cramp, a task-specific upper limb dystonia, patients initially report excessive tightness in hand or forearm muscles—sometimes described as a “cramp” (9). Even though there may be tightness, patients often can still perform the motor task, but over time, motor performance degrades with variable loss of dexterity, fatigue, or even pain. Abnormal postures can occur such as abnormal flexion or extension of the fingers and may be accompanied by abnormal wrist postures as well. It may not always be possible, however, to differentiate between abnormal posture as a manifestation of the dystonia and a compensatory contraction or movement. In this regard, voluntary movement of the contralateral (unaffected) hand may elicit the primary dystonic posture in the affected hand. This is “mirror dystonia,” which refers to a phenomenon in which voluntary movements contralateral to the affected limb provoke dystonic movements on the affected side (10, 11). For instance, in writer’s cramp when writing with the unaffected non-dominant hand, the normal voluntary movement can provoke or cause recapitulation of the dystonic movements in the affected hand, even though it is not engaged in the writing task. Assessing the abnormal posture in the affected hand brought out by “mirror dystonia” may be helpful in selecting the most appropriate muscles for botulinum toxin (BoNT) injection.

Writer’s cramp has been described in the literature since 1830 and was originally classified as one of the “occupational neuroses” (12). Gowers may have been the first to recognize the aspect of “overuse” or repetitive action that typically precedes the development of the dystonic hand posture (12). He described that the abnormal spasm initially occurred only with writing, but later involved other actions—even affecting the non-dominant hand if used for writing (12). Given the relationship to repetitive action, writer’s cramp must be differentiated from overuse syndromes and nerve entrapments (9). Sensory changes, in addition to pain and weakness, may be helpful in identifying peripheral nerve pathology rather than a dystonic etiology as a cause of the symptoms. In general, the remainder of the neurological exam should be normal in writer’s cramp. If abnormalities are found, focal structural lesions as well as neurodegenerative causes of dystonia should be considered (Table 1).

**Table 1:** **Limb dystonia by etiology**.

Isolated: dystonia is the only motor feature	Adult-onset task-specific	Writer’s cramp Musician’s dystonia Runner’s dystonia
	Adult-onset non-task-specific limb dystonia	Idiopathic

Combined: dystonia is combined with other movement disorders	Adult-onset non-task-specific limb dystonia	Parkinson disease Atypical parkinsonian disorder (i.e., corticobasal degeneration) Posttraumatic or complex regional pain syndrome Psychogenic
	Dystonia-plus syndromes	Dopa-responsive dystonia Rapid-onset dystonia parkinsonism Myoclonus-dystonia syndrome
	Paroxysmal dyskinesia and dystonia	Paroxysmal kinesigenic dystonia Paroxysmal non-kinesigenic dystonia Paroxysmal exercise-induced dystonia
	Heredodegenerative dystonia	Wilson’s disease Huntington’s disease Neuroferritinopathy
	Structural lesions	Stroke Tumor

Musician’s dystonia of the hand and arm is a focal task-specific dystonia that classically affects performing artists at the peak of their careers with an average age of onset at 36 years of age (13). Musician’s dystonia can also affect the embouchure. This unusual condition has afflicted famous musicians in the last two centuries, including Robert Schumann, Leon Fleisher, Gary Graffman, Peter Oundjian, and, likely, Yehudi Menuhin (14). Unlike all other forms of focal dystonia, musician’s dystonia of the arm has a striking male to female predilection at 4:1 with prevalence between 1 and 2% of musicians (15, 16). The hand that performs the more complex motor task appears to be preferentially affected (e.g., right hand in pianists, left hand in violinists, right hand in guitarists) (13). Musician’s dystonia can be encountered with essentially all musical instruments, but certain instruments are overrepresented in clinical series of musician’s dystonia, such as keyboard, guitar, and violin. The age at initiation of musical instruction appears to influence risk for development of the disorder, with instruction before age 10 being protective against the development of dystonia.

Typically musician’s dystonia of the hand and arm begins as an insidious deterioration in previously automatic performance, followed by involuntary posturing within months of symptom onset (17). Dystonia may affect the fingers, wrist, upper arm, and even the shoulder girdle. Frequently, the pattern of dystonia segregates with certain instruments, for example adjacent finger flexion in pianists, wrist flexion in percussionists, and shoulder girdle involvement in the bow arm of violinists.

### Current Knowledge Gaps and Areas of Controversy in Upper Limb Dystonia

The epidemiological studies of upper limb dystonia are widely believed to underestimate true prevalence rates, because many cases go unrecognized for many years or they are misdiagnosed as more common conditions, such as repetitive injury syndromes, Parkinson’s disease, or tremor. One of the most common forms of task-specific dystonia is dystonic writer’s cramp, but the prevalence of this form of focal dystonia has not been studied. In fact, one study revealed an average latency of more than 10 years from symptom onset to diagnosis for upper limb dystonias (18). Furthermore, agreement on diagnosis for upper limb dystonias is modest, even among experts (19, 20). The lack of widely accepted diagnostic criteria and reliable biomarkers for upper limb dystonias likely contribute to the poor diagnostic recognition and agreement.

Although there are multiple reports describing the clinical features for relatively large numbers of patients with cervical dystonia and craniofacial dystonia, few address upper limb dystonias. Most reports have included only relatively small numbers of patients with upper limb dystonia or they have focused on specific subtypes, such as writer’s cramp (21–24), musician’s dystonias (see below), or the dystonia associated with Parkinson-related neurodegenerative diseases (25–28).

The cause of musician’s dystonia is obscure, but certainly seems multifactorial with different factors more important in different persons. The settings in which it most often develops involve repetitive performance of a movement that requires great skill. This setting implies the disorder is acquired due to certain environmental factors. While there are some genetic studies that have linked musician’s dystonia in the arm and writer’s cramp with variants in the arylsulfatase G gene, it remains unclear how genetics, environmental influences, and their interactions result in the development of the disorder (29). Classically considered to be an irreversible phenomenon, recent work has raised the possibility that early identification of patients and prompt initiation of treatment might rescue some patients, allowing them to continue their performing careers (30).

Although it is often claimed that 10–15% of patients with idiopathic Parkinson’s disease may present with focal dystonia of the upper or lower limb, especially in early-onset cases, surprisingly few studies report the prevalence of this phenomenon, or of the clinical characteristics, that help to distinguish these cases from non-degenerative adult-onset focal limb dystonia (31, 32). The paucity of large clinical studies comparing the clinical features distinguishing the limb dystonias of degenerative Parkinson-related disorders from the limb dystonias of non-degenerative adult-onset isolated focal dystonias likely contributes to frequent misdiagnoses. Indeed, multiple studies have described patients with isolated limb dystonia who were misdiagnosed as having Parkinson’s disease (33–36).

Some patients exhibit semi-rhythmical movements of the hand and arm, with little or no postural abnormality. When these movements occur only with writing, they are often called primary writing tremor. It remains controversial whether these types of abnormal movements should be classified as a subtype of dystonia (e.g., dystonic tremor), as a subtype of essential tremor, or as a distinct entity (37–44). Without a reliable biomarker for either dystonia or essential tremor, the exact classification will remain a matter of debate.

### Dystonia of the Lower Limb

Focal or segmental dystonia confined to the lower limb is an uncommon focal dystonia and requires a meticulous assessment and testing to exclude other conditions, such as parkinsonism, stiff-person syndrome, and other movement disorders (45, 46). Runner’s dystonia (RD), an important but often undiagnosed or misdiagnosed type of lower limb dystonia, is defined as a task-specific focal or segmental dystonia of the lower limb or trunk triggered by running (47). Patients with RD often describe their initial symptom as a subtle change in their gait or running stride, a limp or a sense of pulling, cramping, or stiffness triggered by running and improved with rest. At first, they often attribute their symptoms to overuse, a change in shoes or a different running surface. They may also suspect “foot drop,” an injury (muscle strain/sprain), or other musculoskeletal complaint (48). Commonly reported symptoms in RD include a limp when running, dragging of the foot or leg, inversion of the foot, scuffing of the toe, clipping an ankle with the opposite foot, trunk tilt, and/or pain. Similar to other focal dystonias, patients with RD may report an alleviating maneuver (also referred to as “geste antagoniste” or “sensory trick”), which improves their symptoms (49, 50).

When symptoms persist or worsen, a patient commonly self-refers to an athletic trainer/coach, physical therapist, sports medicine, or orthopedic physician—delaying the correct diagnosis often by many months or even by years (51). A missed diagnosis may also lead to unnecessary therapies and/or invasive procedures (52). By the time a patient with RD consults with a movement disorder specialist, their symptoms have often generalized to involve walking, and running may be limited or impossible. A possible clinical clue to the diagnosis of RD is a marked improvement in, or complete absence of, symptoms when the patient walks or runs backwards (i.e., task specificity).

Assessment of patients with suspected RD includes a history and physical examination with special attention to the musculoskeletal and neurological systems. The differential diagnosis of RD includes a focal dystonia presenting as the initial symptom of primary generalized dystonia, a secondary process (stroke, Parkinson’s disease), trauma, and functional (psychogenic) causes (45, 53, 54). If not previously performed, the diagnostic work up may include electrodiagnostic testing, spine/brain/skeletal imaging, and laboratory studies including metabolic and, potentially, genetic testing. Functional assessment in RD includes observational and videotaped assessment of the patient at rest, standing, walking, and running. Video assessment may reveal subtle findings that are missed during real-time observation and can be used to evaluate the response to an intervention.

When RD is suspected and questions remain about the diagnosis after history and clinical exam, 3D computerized motion analysis may provide useful information about which muscles are involved. This method may help identify specific causes for this difficulty and guide treatments (55). Careful analysis of the electromyography (EMG) data is required paying special attention to the timing and duration of muscle activation, the relationship to kinematics, and side-to-side comparison. Abnormalities of muscle activation in patients with dystonia include onset, timing, duration, magnitude of recruitment, depression or prolongation of phasic bursts, and co-contraction; however, no studies have proven these tests to be diagnostic. Other abnormalities considered to be consistent with dystonia include activity at rest, an inability to relax when a movement ends, and overflow to an unwanted body part.

### Current Knowledge Gaps and Areas of Controversy in Lower Limb Dystonia

Lower limb dystonia is less common than other focal dystonias, such as cranial and cervical dystonias, but its true prevalence is not known and is likely currently underestimated. Clinical features such as task specificity and the use of sensory tricks can be seen in lower limb dystonia, similar to other forms of dystonia. The relationship between isolated leg dystonia and other neurodegenerative diseases (i.e., Parkinson’s disease) is not well understood.

### Key Research Priorities in Clinical Features of Upper and Lower Limb Dystonias

- Development of clinical diagnostic criteria for upper and lower limb dystonias, taking into consideration their clinical heterogeneity
- Clarify the relationship between dystonia, tremor, and dystonic tremor
- Clarify the relationship between dystonia and mirror dystonia
- Systematic characterization of clinical characteristics of patients presenting with isolated limb dystonia who are likely to progress to Parkinson’s disease or a related degenerative parkinsonian condition
- Characterize the genetic and environmental influences on the development of musician’s dystonia

### Posttraumatic Dystonia, Peripherally Induced Dystonia, and Complex Regional Pain Syndrome (CRPS)

Central (brain) trauma has been long recognized as a cause of dystonia, but peripherally induced dystonia, triggered by trauma to the cranial or peripheral nerves or roots, is still controversial (56). In a review of 190 articles presenting findings on 596 patients with peripherally induced movement disorders, the most frequently reported movement disorder was dystonia (74%), followed by tremor (23%), myoclonus (15%), spasms (11%), painful limbs moving extremities (6%); and another 2% had parkinsonism, chorea, and tics (57). Most studies reported latencies of less than 1 year (median = 21 days), but in 27 cases (5%) the reported interval between injury and the onset of movement disorder was greater than 1 year. Only 170 patients (29%) showed evidence of a nerve injury. Pain was an important feature in the majority of patients (81%) and preceded the onset of movement disorder in 20% of the cases. CRPS was diagnosed in 42% of the reported cases but only 8% had nerve injury. BoNT was the most frequently applied therapy (21%) in this review, with 57% of patients treated with BoNT reporting mild or moderate improvement in symptoms. Physical therapy and oral medications, such as trihexyphenidyl, baclofen, and muscle relaxants, provide only limited benefit in this population. Due to a concern of abnormal sympathetic drive in this disorder, chemical and surgical sympathectomies have been used in this patient population, but due to common complications after sympathectomy and lack of evidence of clear long-term benefit, it is now rarely used (58). Deep brain stimulation (DBS) has been only rarely reported to be beneficial in patients with peripherally induced dystonia (59).

**Figure 1:**
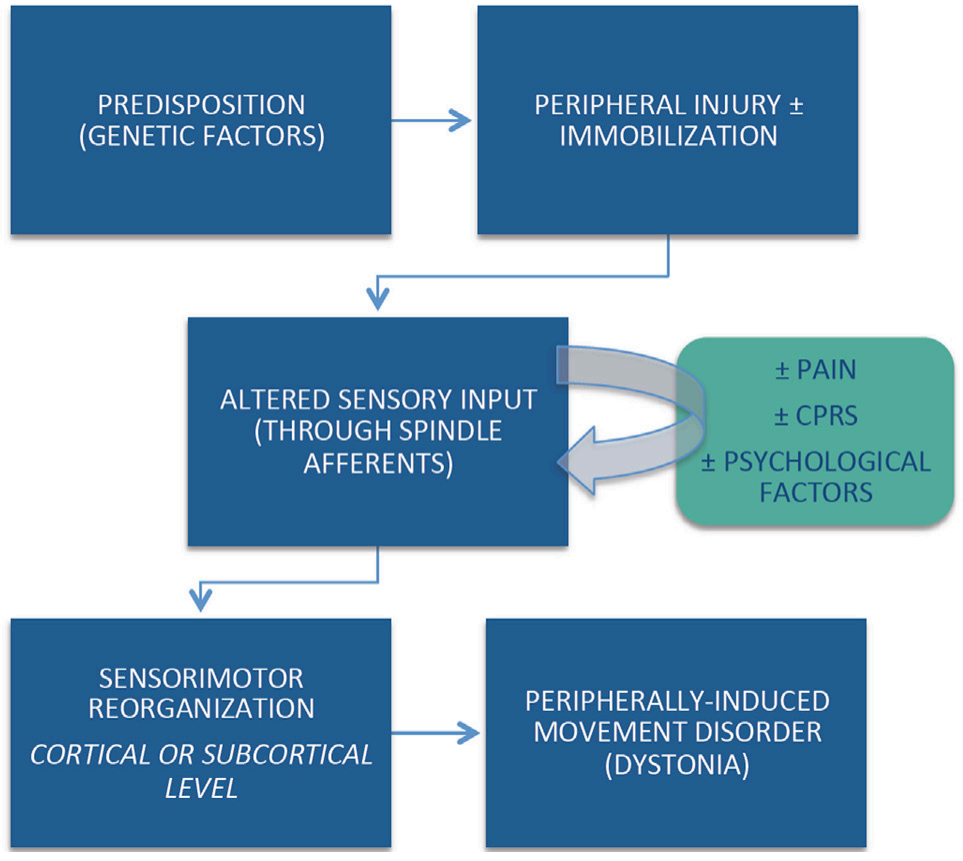
**Diagram of hypothesis of peripherally induced movement disorders**.

Despite strict diagnostic criteria, including the requirement for anatomically and temporally related injury, the cause-and-effect relationship between the peripherally induced injury and subsequent movement disorder may not be obvious in all cases. Although the pathophysiological mechanisms of peripherally induced movement disorders are not well understood, emerging evidence suggests that individual (e.g., genetic) predisposition, coupled with central reorganization in response to the altered peripheral input, plays an important role in the pathogenesis of peripherally induced movement disorders (Figure 1). Arm immobilization, a form of peripheral injury, can lead to decreased thickness in the contralateral primary motor and somatosensory cortical area and a decrease in the white matter fractional anisotropy in the contralateral corticospinal tract (60). Cortical reorganization in the primary sensorimotor cortex occurs following arm amputation (61). Abnormal activation on functional magnetic resonance imaging (fMRI) in regions such as the basal ganglia and other brain regions reported in patients with CRPS have not been confirmed by other studies (62). This may be partly explained by heterogeneous population of patients, small sample size, and methodological issues related to fMRI (63).

### Current Knowledge Gaps and Areas of Controversy in Peripherally Induced Dystonia

One of the major sources of debate related to peripherally induced dystonia is its possible relationship to functional (psychogenic) movement disorders. This controversy is particularly highlighted by the phenomenon of “fixed dystonia.” In the classic report by Schrag et al., the authors described the clinical features of 103 patients presenting with fixed dystonia, primarily (90%) involving the limb (64). They followed 41 patients prospectively for a mean of 3.3 years. In 63% of patients, the dystonia was preceded by a peripheral injury and in 56% the dystonia spread to other body regions. During the follow-up period, only 27% achieved partial or complete remission. Pain was a major complaint in 41% of the patients, and 20% met the criteria for CRPS. Although only 37% of the patients fulfilled diagnostic criteria for “documented or clinically established psychogenic dystonia,” the authors concluded that “many patients fulfill strict criteria for a somatoform disorder/psychogenic dystonia” and that fixed dystonia “usually, but not always, occurs after a peripheral injury and overlaps with CRPS” (64). Other studies have failed to establish direct connection between CRPS and an abnormal psychological profile (65). Many patients with CRPS, however, share demographic and clinical features with those diagnosed as functional (psychogenic) movement disorders, such as female preponderance, young age, and abrupt onset (64). Although no specific abnormalities in brain structure or function have been consistently identified in patients with CRPS, it would be premature to conclude that CRPS is a functional (psychogenic) disorder (62).

In addition, there are other controversies concerning the diagnosis and the pathophysiology of peripherally induced or posttraumatic dystonia. Besides peripheral injury, prolonged immobilization seems to be an important risk factor. One distinguishing clinical feature from other dystonias is the frequency of pain as a presenting complaint. Although local injections of BoNT into the muscle of the dystonic limb or an intradermal injection in the region of the pain may improve the motor and sensory aspects of CRPS-related dystonia, therapeutic options for this disorder currently are limited and have not been systematically studied to date (66).

### Key Research Priorities in Peripherally Induced Dystonia

- Systematic clinical and neurophysiological characterization of patients with peripherally induced dystonia compared to focal, idiopathic limb dystonia, and to healthy controls
- Investigation of patients with pre-existing dystonia following peripheral injury and/or immobilization using epidemiologic, phenomenologic, neurophysiologic, and imaging studies to identify any factors that might exacerbate underlying dystonia to provide insights to peripherally induced dystonia
- Development of a suitable animal model that can explore the effects of peripheral injury on spinal cord and brain and its role in centrally mediated dystonic symptoms
- Design and conduct double-blind, controlled clinical trials of BoNT in patients with peripherally induced dystonia to establish the level of evidence that this treatment modality is safe and effective in this population

## Pathogenesis

### Inhibition in Dystonia: Motor and Sensory

The pathophysiology of dystonia is characterized by a loss of inhibition, which has been shown at multiple levels in the nervous system, from the spinal cord to the brainstem to the motor and sensory cortical regions (67). This loss of inhibition manifests in the periphery with abnormally long muscle bursts as measured by EMG, co-contraction of agonist and antagonist muscles, and overflow into adjacent muscles not needed for the particular motor task (67).

Several measures of cortical excitability examined in focal limb dystonia have revealed abnormalities including short intracortical inhibition, mediated through GABA-A receptors, and long intracortical inhibition, mediated by GABA-B (68). Another measure of cortical excitability is the cortical silent period (CSP). The CSP is a pause occurring during voluntary movement after a pulse of transcranial magnetic stimulation (TMS) is applied to the contralateral motor cortex. There are both spinal cord and cortical inhibitory contributors to the CSP, the latter likely mediated through GABA-B receptors (69, 70). In writer’s cramp, the CSP is shortened compared to controls, indicating an overall loss of inhibition in the motor system (71). Interestingly, this finding has been seen only in the symptomatic hand and not in the asymptomatic side. Further specificity was seen in a study of writer’s cramp where the CSP was significantly shorter in the patient group only during a pincer grasp but not during a power grip condition, suggesting some task specificity in this abnormality (72). During a pilot trial examining individualization of therapeutic repetitive TMS in two focal hand dystonia patients, one of the response variables used was the CSP (73). The investigators found that the subject with the shortened CSP responded favorably to the repetitive TMS (rTMS) and had both a physiological response with lengthened CSP and a subjective clinical improvement (73).

While as noted above, the pathophysiology of focal dystonia has generally shown a loss of inhibition, there are some examples in the literature of enhanced inhibition in dystonia patients in particular cortical pathways. A dorsal premotor to primary motor cortex abnormality has been identified in writer’s cramp patients at rest, where the writer’s cramp show enhanced inhibition compared to healthy controls (74). This inhibitory influence from the premotor cortex (PMC) was found to be supraspinal in nature, as the H-reflex did not change with premotor conditioning. Evaluating this abnormal premotor–motor interaction through a biomarker analysis showed an area under the curve of 0.825 with a sensitivity of 84% and specificity of 74% (74). Whether this abnormality is a primary manifestation of the disease or is a compensatory change is not clear, but a pilot trial enhancing inhibition over the PMC in cervical dystonia has shown some promise (75).

Abnormalities in inhibition in the sensory system have also been identified in focal dystonia—specifically in the somatosensory temporal discrimination threshold (STDT). The STDT is the shortest time interval necessary for a pair of tactile stimuli to be perceived as two (76). STDT has been shown to be abnormal in dystonia, including focal hand dystonia (77, 78) and in cervical dystonia (79). However, abnormalities in STDT are not specific for dystonia as they may be seen in other patient populations (e.g., Parkinson’s disease) (80). The pathophysiology of abnormal STDT has been demonstrated to be due to a loss of a short latency inhibitory process (78). Using inhibitory non-invasive neurostimulation, the STDT was increased in healthy volunteers (81). This led to the clinical effect of overall decreased ability to discriminate between paired inputs, suggestive of at least *part* of the phenotype seen in dystonia. This may be an instructive tool to improve our interpretation of abnormal STDT and recapitulate part of the phenotype in a human “model.”

### Plasticity in Dystonia

Another theme that has emerged in the pathophysiology of focal dystonia is aberrant cortical plasticity. One widely used method to assess cortical sensorimotor plasticity is paired associative stimulation (PAS). Repeated pairs of peripheral nerve stimulation, typically median nerve stimulation at the wrist, followed by TMS of the motor cortex (M1) between 21 and 25 ms later, induce cortical plasticity. This produces a spike-timing dependent, long-term potentiation (LTP)-like plasticity at the level of the M1, also known as associative plasticity. Initial studies in patients with writer’s cramp showed excessive plasticity with abnormal spread of the induced plasticity to non-targeted muscles (82, 83). The increased LTP-like plasticity extends to body parts unaffected by dystonia. For example, patients with cervical dystonia, blepharospasm, and oromandibular dystonia, all had excessive plasticity measured in their unaffected hand muscles (84).

The ability to regulate plasticity to keep excitability within a useable range, known as homeostatic plasticity, is also impaired in dystonia (85). In addition to examining motor cortical plasticity, studies have also measured somatosensory-evoked potentials and found increased LTP-like plasticity in the somatosensory cortex in patients with focal hand dystonia (86). Taken together, these studies lead to the attractive hypothesis that task-specific hand dystonia is related to excessive plasticity, possibly due to abnormal association between sensory input and motor output with deficient homeostatic control (87).

It should be noted, however, that several studies did not find increased sensorimotor plasticity in focal hand dystonia using PAS, and the results from different studies have varied (88). Some of these conflicting results may be due to the inherent variability of the effects of PAS even in healthy subjects (89, 90). In addition, there is also variability in the PAS paradigms resulting in differing times between median nerve stimulation and TMS (e.g., 21.5 vs. 25 ms) (91). Other factors including the repetition rate, the stimulus strength and the number of paired stimuli delivered, the state of muscle activity, the time of the day, attention to the stimuli, and genetic factors, all are important factors in measurements of plasticity (92). Another form of LTP-like cortical plasticity induced by intermittent theta burst stimulation, which does not involve sensory input, showed abnormal plasticity but was *decreased* rather than increased in focal hand dystonia (93). These findings suggest that abnormal processing of sensory input may underlie increased associative plasticity in focal hand dystonia, but the direction of change is variable depending on the study paradigm and the exact part of the sensorimotor cortex probed.

One way to understand the role of plasticity in dystonia is through the relationship between associative plasticity and the effects of DBS, a therapy used to treat generalized dystonia and less often, focal dystonia. DBS targeting the globus pallidus interna (GPi) decreases excessive associative plasticity in patients with generalized dystonia (94). However, the time of the maximum decrease in plasticity occurred before the subsequent, maximum clinical improvement, raising the possibility that the reduction in excessive plasticity may drive clinical improvement (94). Moreover, in patients with generalized dystonia who had the DYT1 gene mutation, the degree of associative plasticity correlated with the maintenance of clinical benefit after GPi DBS was turned off for 2 days (95). In these patients, associative plasticity seemed to reflect an ability to store normal movements and to resist abnormal signals from the basal ganglia even while the therapy was turned off. Direct measurement of GPi activity during DBS implantation has also provided evidence that short-term plasticity is abnormal in dystonia patients, with impaired paired-pulse depression seen (96). This and other studies suggest that the impaired inhibition seen cortically in associative plasticity studies is also reflected at the basal ganglia level in direct recordings (96–98).

### Task Specificity

One of the most fascinating features of limb dystonia is task specificity. This refers to the situation where dystonia is manifested only during a single task or several closely related tasks, such as in writer’s cramp and in musician’s dystonia (e.g., pianist’s cramp is only manifested when playing the piano). Although more common in the upper limbs, task-specific dystonia can also affect the face (e.g., embouchure dystonia) and the leg (e.g., RD). As discussed earlier, the dystonia appears to be triggered, at least in part, by repetitive skilled action, and virtually any task can be affected. At onset, the dystonia can be very highly selective; some cases of writer’s cramp, for example, have begun with involvement of only a few specific letters. Moreover, the dystonic posture can be highly focal involving only one or two fingers. While many patients with a task-specific dystonia remain with relatively restricted involvement, the dystonia can spread to involve more muscles, becoming segmental dystonia or even more generalized dystonia. In some patients, the task specificity is gradually lost with dystonia affecting more tasks or even appearing at rest.

Why does repetitive activity drive the development of a task-specific dystonia? Much evidence suggests that repetition, in-and-of-itself, is not the sole driver, but that it is the interaction of repetitive activity with multiple factors. One likely factor is a genetic predisposition. Another is inherent biomechanical abnormality of the hand (99). If the hand is anatomically abnormal, then the motor control program might require modification in order to accomplish the intended motor task. Another critical factor seems to be abnormal plasticity processes in the brain. As noted above, good evidence suggests that patients with limb dystonia have abnormal homeostatic mechanisms to control the upper and lower bounds of plasticity as well as heightened plasticity overall, which is widespread both anatomically as well as within the different dystonia types (100). The abnormalities of plasticity suggest an endophenotype, not necessarily the cause of dystonia by itself, but predisposing to the development of dystonia. The combination of repetitive activity, heightened plasticity, and failure of limiting plastic change may well be the particular combination needed to drive the development of dystonia.

How is it possible to have a task-specific deficit? Considering writer’s cramp, for example, motor control in the hand itself is basically working since all actions except writing are done well. Moreover, the motor program for writing remains intact since writing can be done normally with other limbs, albeit somewhat clumsily. Hence, task specificity arises just with the particular conjunction of a specific limb with a specific task. The pattern of brain activation with a specific body part is well established with somatotopic involvement of the primary motor cortex, cerebellum, and lateral and medial PMC. The motor program for writing has also been studied and includes parts of the PMC and parietal areas. A special area in the PMC concerned with writing is Exner’s area—near and analogous to Broca’s area for speech. A specific subset of the overlap between these two regions must be responsible for writing with the dominant hand, the usual limb for writing and, therefore, the body part at risk for the development of dystonia. In an fMRI experiment to determine task-specific activation, stronger activations in the left dorsal prefrontal cortex, left intraparietal sulcus, and right cerebellum in writing were found compared with all other tasks. Additionally, the left anterior putamen was active at onset for all the tasks, but only showed sustained activation during the right-hand writing. An exploratory analysis showed clusters in the left ventral PMC and inferior and superior parietal cortices that were only significantly active for right-handed writing (101).

A similar experiment was conducted in patients with writer’s cramp. The regions that were task-specific in the normal individuals were less active in patients. Moreover, the connectivity between the parietal and premotor areas was less strong (102). Hence, it appears that a specific parietal–premotor pathway was malfunctioning. In some sense, this is not surprising. Individual parietal–premotor pathways do seem specialized for specific tasks. This has been demonstrated most clearly for a reach-to-grasp movement, where there are separate pathways for each component (103). Moreover, large lesions of either parietal or premotor areas will cause apraxia with a loss of many skilled movements (104). Thus, a task-specific deficit could arise from the interaction of a pathway where a specific task was learned together with excessive motor repetition of that particular task in the setting of uncontrolled plasticity.

### Functional Imaging of Limb Dystonia

Functional imaging in isolated limb dystonia has helped to identify underlying pathophysiologic mechanisms, as exemplified in the previous section. Various functional neuroimaging methods have been used with other goals in mind and include molecular imaging focusing primarily on brain hemodynamics or changes in dopaminergic pathways and fMRI of resting-state blood oxygen level dependent signals.

Positron emission tomography (PET) measures of regional cerebral blood flow can identify local blood flow responses to various stimuli. The general strategy has been to measure blood flow with the participant at rest in the scanner and then repeat the PET measure during an activation procedure. Local changes in either blood flow or metabolism reflect local neuronal activity or the changes in activity of terminal fields projecting to that area (105). Initial studies of brain responses to hand movements in people with isolated upper limb dystonia revealed differences in blood flow responses. However, differences in how someone with hand dystonia and a control subject move the hand could substantially confound interpretation of such studies. This methodologic ambiguity and the observation of sensorimotor integration problems in people with hand dystonia led to studies of blood flow responses to sensory driven stimuli.

Vibration of a hand produces a blood flow response in contralateral sensorimotor cortex and supplementary motor area. People with isolated hand dystonia, including a subgroup with only right-handed writer’s cramp, show an approximately 25% reduction in these blood flow responses (106, 107) similar to findings in other isolated dystonias (108). The vibratory stimulus elicited a cramp in some of the dystonic participants, but these participants did not have a different blood flow response from those who did not have cramping. The healthy controls who simulated a dystonic posture during the vibratory stimulus had an increased, rather than decreased blood flow response. Similarly, a patient with dopa-responsive dystonia showed reduced blood flow response to vibration that normalized after a dose of l-DOPA (109). This observation suggested that the vibration-induced blood flow responses could be influenced by dopaminergic pathways.

Positron emission tomography also can provide direct measures of dopaminergic receptors with most studies finding a reduction in D2-like dopaminergic receptors. MPTP, a neurotoxin selective for dopaminergic neurons, when given *via* one internal carotid artery in non-human primates, produces contralateral transient limb dystonia followed by chronic parkinsonism (110, 111). During the transient dystonic phase, striatal D2-like receptor binding is reduced about 25–30% but then increased several fold during early parkinsonism. The increased D2-like receptor binding gradually returned toward normal. However, mRNA selective for D2R (selective for D2R over D3R) revealed no change whereas mRNA for D3R did increase coinciding with the D2-like receptor changes (112). These findings presaged studies in humans with isolated limb dystonia that revealed a similar reduction in striatal D2-like binding in those with either isolated, idiopathic hand, or cranial dystonia (113–115). In fact, the site of change in the putamen seems to relate somotopically to the part of the body involved (116). Some have used [^11^C]raclopride as the D2-like radioligand. This particular radioligand can be displaced by increased release of endogenous dopamine. This characteristic has permitted measures of striatal dopamine release in response to drugs or tasks. In particular, a finger-tapping task elicited less dopamine release (measured as a change in striatal uptake of [^11^C]raclopride) in people with writer’s cramp whereas a speech task in those same subjects yielded greater striatal dopamine release (115). Key findings from these studies is that striatal D2-like receptor binding is likely abnormal in limb dystonia and changes in dopamine release may also occur.

The selectivity of these changes for specific D2-like dopamine receptors remains unclear. PET measures with a D2 highly selective radioligand [^18^F]*N*-methyl benperidol (D2 ≫ D3 selectivity) did not reveal any changes in people with either hand or cranial dystonia (117). This suggests that the findings with less selective D2-like radioligands may reflect a change in D3 dopamine receptors, which would be consistent with the observation in MPTP-induced transient dystonia in monkeys (110–112); however, confirmation of this notion awaits development of a highly selective D3 radioligand for PET. Nevertheless, D1-like dopamine receptors appear to be normal in hand and cranial dystonia (118). Thus, these studies indicate a change in dopamine receptors possibly due to a change in striatal D3 specific dopamine receptors in dystonia patients.

At this point, the focus has been on neuroimaging findings that relate to changes in striatal function or activity. Yet, increasing data suggest that the dystonia also may reflect changes in cerebellar function that may result from either direct involvement of cerebellum by functional connections with other brain regions or networks. Resting-state functional connectivity studies with magnetic resonance imaging (rs fcMR) have demonstrated strong functional connectivity in humans between striatum and a large area extending from upper and middle brainstem into cerebellum (119). These findings do not necessarily reflect direct anatomic connections but evidence for direct connections in non-human primates between the cerebellum and the subthalamic nucleus *via* the pons (120). The cerebellar vermis also has direct connections to primary motor and premotor areas (121)—areas that also have functional connectivity with the striatum. Thus, dysfunction in a brain network, either precipitated by direct involvement of a specific node or modulation at the network level, may provide the underlying pathophysiology of limb dystonia.

In support of this notion, rs fcMR studies indicate reduced functional connectivity between inferior parietal lobule and dorsal PMC contralateral to right-handed writer’s cramp patients (122). Another rs fcMR studied revealed increased functional connectivity with the left putamen as a component of the default mode network (DMN) in 16 people with right hand writer’s cramp compared to controls. Although the putamen is not typically considered part of the DMN, a network that includes prefrontal, anterior and posterior cingulate, lateral parietal, inferior and middle temporal area, cerebellar areas, and thalamus (123), the comparison of the independent component containing the DMN between the writer’s cramp and control groups revealed this increased putamen functional connectivity (124). In this same study, the writer’s cramp group had reduced functional connectivity with the left PMC that was part of the sensorimotor network. Both of these findings were affected by BoNT injections. The advantage of these resting-state studies is that they are not confounded by either behavioral changes during the scanning sessions or performance differences that could differ between those with limb dystonia and control groups.

### Current Knowledge Gaps and Areas of Controversy in Dystonia Pathogenesis

The pathophysiology of focal limb and task-specific dystonia is characterized by a loss of inhibition, impaired sensorimotor integration, and aberrant cortical plasticity as seen through non-invasive neurostimulation studies. How precisely task specificity emerges from these underlying neurophysiologic changes is not known; but, factors such as an underlying endophenotypic trait of abnormal plasticity combined with repetitive task-specific movement generated in a particular sensorimotor network—all appear relevant. This issue is a key area to focus on moving forward in order to clarify how plasticity abnormalities translate into the clinical expression of dystonia. In addition, functional neuroimaging studies have revealed changes in dopaminergic pathways in the striatum and altered striatal and cerebellar pathways in dystonia patients. Together, these various findings suggest that changes at the network level underlie limb dystonia and raise questions about whether cortical–striatal–thalamo cortical networks are really segregated from cerebellar–thalamic–cortical networks. In particular, the relationship with the dopaminergic system in dystonia is intriguing and worthy of future exploration. This includes further invasive and non-invasive paired-pulse studies (such as in GPi DBS for dystonia). As mentioned earlier, the relationship between isolated focal limb dystonia and posttraumatic, peripherally induced dystonia is unclear. There have been several studies aiming to explore at this question. A recent study looking at patients with a fixed hand posture and CRPS compared to healthy controls found sensorimotor abnormalities potentially compatible with a psychogenic dystonia and in contrast to findings found in isolated focal hand dystonia (125). Identifying similarities and contrasts between the underlying pathophysiology of these disorders will be helpful moving forward perhaps both in diagnosis and in treatment.

### Key Research Priorities in Limb and Task-Specific Dystonia Pathogenesis

- Further studies to distinguish cause from effect in both physiology and imaging studies, so that attention can be directed to the most relevant biological correlates of dystonia
- Development of a diagnostic battery using neurophysiologic and imaging tests, including identifying whether one test will be sufficient for all focal dystonias
- Identification of therapeutic targets
- Understand the variability and reproducibility of PAS and other non-invasive measurement tools in healthy subjects and dystonia patients and standardization of study protocols to minimize variability across studies
- Determine how exactly abnormal plasticity affects the specific parietal–premotor pathway and how this relates to spread of dystonia beyond a particular task or limb

## Therapy

### BoNT for Treatment of Limb and Task-Specific Dystonias

Botulinum toxin has a well-recognized role in the treatment of limb and task-specific dystonias; although, the amount of Level I evidence available is limited (126). Currently, three BoNT type A formulations (onabotulinumtoxinA, abobotulinumtoxinA, and incobotulinumtoxinA) are approved for upper limb spasticity and only one, onabotulinumtoxinA, for lower limb spasticity (127). None of these are approved, however, specifically for focal limb dystonia.

Several randomized, double-blind, controlled studies in limb dystonia have been performed investigating abobotulinumtoxinA (128, 129) and onabotulinumtoxinA (130–132). When comparing these studies, outcome measures and populations enrolled have marked variability. This highlights one characteristic of focal limb dystonia that makes obtaining reliable data on efficacy challenging. Standardized scales or outcome measures capturing all types of task-specific or limb dystonia are lacking. In addition, the very nature of task specificity makes it difficult to generalize, and its clinical manifestation and prevalence tend to change with occupational skills relevant to the era and to the particular society (133). The impact on quality of life is very patient-dependent, and treatment response is at times radically different from other conditions responsive to BoNT therapy (134). Another area of interest is the choice of toxin for specific indications. To date, no Level I studies have been performed allowing a comparison of available formulations for limb and task-specific dystonia. Comparative studies have been conducted in blepharospasm and cervical dystonia populations, but it is not clear to what extent the results can be extrapolated to focal limb dystonia.

Regarding injection technique, accurate targeting of the relevant muscles, and avoidance of toxin spread to adjacent structures are clearly desirable. Little data are available, however, on the best guidance tools among the available options. There is some evidence that using a guidance method, such as EMG, is superior in accuracy to anatomic guidance alone (135) but it is not clear how this translates into efficacy. This issue of efficacy has been studied in limb spasticity, comparing electrical stimulation and ultrasound guidance (136, 137) but not in limb dystonia. Studies are ongoing comparing guidance techniques in this patient population (Clinical Trials identifiers NCT02334683; NCT02326818), and more are needed.

### Rehabilitation Interventions for Limb Dystonias

Given the sparse literature on the topic and the rarity of the disorder, there are no clinical practice guidelines on rehabilitation in upper or lower limb dystonia. However, conventional rehabilitation methods, such as stretching, strengthening exercises, manual therapy, and splinting programs, are frequently used in clinical settings when patients are referred for physical or occupational therapy. These therapies are also often tested as a control intervention or combined with other therapies in research investigating efficacy of a novel intervention protocol (138–140).

In limb dystonia patient populations, investigators have proposed various forms of intensive motor training to recover voluntary motor control. These approaches have been frequently explored in treatments of musician’s dystonia using the methods known as “slow down therapy” and “sensorimotor retraining” (141). Other approaches have prioritized the reorganization of the cortical somatosensory map using methods, such as Braille training (142, 143), “learning-based sensorimotor training” (138, 139, 144), or prolonged immobilization of the affected limb (this method is no longer used) (145, 146). Attempts to normalize muscle activity to restore voluntary control using biofeedback, vibration, or electrical stimulation have also been used (147–151). Similar to constraint-movement therapy, a method often used in stroke rehabilitation, some investigators have used motor practice combined with constraining the unaffected joints with the goal of decreasing compensatory movements (152, 153). Finally, combining neuromodulation methods with motor training in an attempt to normalize brain excitability and further improve motor performance has been tried either with transcranial direct current stimulation (tDCS) (154–156) or with rTMS (140).

Despite the different theoretical bases of the interventions, when considered together, rehabilitation studies in limb dystonia suggest positive outcomes (157–159). Significant improvements have been reported in rating scales of dystonia severity, arm disability, quality of musical performance, and quality of life (140, 144, 153, 160, 161). Studies that focused on sensory reorganization have reported increase in sensory discrimination (138, 139, 142, 144). Furthermore, improved motor performance in writing, gait, and musical performance has also been reported (142, 153, 161, 162).

Limitations of the above studies are typical of small-scale trials and include lack of control groups, blinding, or randomization. It is also likely that the interventions tested were of insufficient duration, considering that limb dystonia likely develops over a long period of time. As the rehabilitation research in limb dystonia develops, it will be important to investigate comparative effectiveness of interventions to understand which approach holds the most promise and the neurophysiological mechanism of effect. Given the nascent stage of rehabilitation research in focal dystonias, full-scale clinical trials have yet to be conducted. Thus, definitive statements cannot yet be made regarding efficacy and clinical implementation of a particular methodology.

A major challenge of rehabilitation intervention studies in general is determining an appropriate control and clearly specifying the interventions to improve reproducibility. The hallmark of rehabilitation is that it involves active participation by the patient and is tailored to each patient’s unique need, which can create problems for reproducibility. For a control to be effective, it must be believable as a true intervention but not contain the key components of the experimental condition. Indeed, blinding and control are essential for future studies, as care from a therapist may impart benefits secondary to feeling cared for in addition to a pure placebo effect. Consequently, future investigations in dystonia need to carefully address this issue by comparing different treatment strategies with similar frequency, duration, and interaction between patient and therapist.

Study designs in a rare and heterogeneous disorder, such as dystonia, require careful consideration outside of the gold standard multisite, randomized controlled trial. Small-scale trials are appropriate given our limited understanding. However, studies should utilize robust small *n* methodology such as single subject experimental design studies with repeated measures (163).

### Non-invasive Brain Stimulation and Hand Dystonia

Non-invasive brain stimulation techniques, such as rTMS and tDCS, have been applied in both basic research into the pathophysiology of hand dystonia and in therapeutic trials (164). Both methods can alter brain excitability in sensorimotor networks, which can be used to reduce abnormal excitation in sensorimotor cortex. The precise neurophysiological mechanisms underlying this change in excitability are not fully understood; however, high frequency rTMS and anodal tDCS are able to increase excitability of the sensorimotor cortex (165). Low-frequency rTMS and cathodal tDCS achieve excitability changes in an inhibitory direction (165). The effects of non-invasive neurostimulation are far more complex than unidirectional excitability change and are not limited to the site of the stimulating electrodes but extend to frontal and parietal networks as well as to the basal ganglia and to the cerebellum (166, 167).

Several small controlled therapeutic trials of writer’s cramp have been done with inhibitory low-frequency rTMS (164). Studies typically included less than 10 mostly writer’s cramp subjects and most used a crossover design with a single session of stimulation targeting the contralateral hemisphere to the dystonic hand. Siebner et al. found that M1 stimulation modestly improved focal hand dystonia (168); however, Murase et al. showed that PMC was a better target than M1 and supplementary motor area to reduce writer’s cramp symptoms (169). Subsequently, more studies used PMC as the target in multi session interventions and showed promising results either by physiologic or behavioral measures (170, 171). rTMS combined with sensorimotor retraining did not provide objective improvements in patients despite subjective improvement in six of nine (73). The results of these small-scale clinical trials with low-frequency rTMS have been mixed, and it is not currently ready for clinical application in this population.

In the last years, tDCS has gained popularity, partly due to its simple application combined with its low cost and low risk for adverse events. In patients with musician’s dystonia (e.g., professional guitarists), a single session of cathodal tDCS targeting the affected M1 did not improve the performance of guitar playing (172). Similarly in pianists, a single session of cathodal or anodal tDCS of the affected M1 combined with simultaneous retraining consisting of slow, voluntarily controlled movements on the piano did not result in any improvement in dystonia (173). The same strategy did not help patients with writer’s cramp (174). In contrast, cathodal tDCS of the affected M1 and simultaneous anodal tDCS of the unaffected M1 in dystonic pianists improved the rhythmic accuracy of sequential finger movements with the affected hand, but only if concurrent bimanual mirrored finger movements were performed (155). This improvement lasted for 4 days after the intervention. Neither a reversed montage of electrodes (anodal tDCS of the affected M1, cathodal tDCS of the unaffected M1) nor unilateral anodal tDCS of the unaffected M1 or sham stimulation yielded any improvement (155). Furthermore, the amount of motor improvement correlated directly with the severity of the symptoms, that is, the most severely affected patients benefited most from the intervention. These findings suggest therapeutic potential in behavioral training assisted by bihemispheric and polarity-specific tDCS in restoring fine motor control in musician’s dystonia. A further single-case study showed augmented therapeutic effects through bihemispheric tDCS combined with bimanual mirrored retraining over two successive days (175). Another group explored biparietal tDCS during neurorehabilitation and showed improvement in dystonia severity in musicians (156).

### DBS and Limb Dystonia

Deep brain stimulation targeting GPi is a highly effective treatment for medically refractory isolated generalized dystonia, supported by high quality case series and randomized controlled trials (176). Patients with cervical dystonia who respond neither to medications nor targeted injections of BoNT may also benefit from DBS, but less reliably so (177). In contrast, the experience treating focal limb dystonia with DBS is quite sparse, most likely because this form of dystonia is uncommon and rarely debilitating, so that the potential risks of DBS surgery seem unwarranted. On the other hand, task-specific dystonias may force an individual to forego an activity that makes his or her life meaningful and BoNT injections can yield significant weakness in both the treated and adjacent muscles, denying the individual the fine motor skills required to perform the practiced task despite relief of their abnormal dystonic posture.

A review of the literature regarding stereotactic surgery for focal limb dystonias reveals the following: (1) fewer than 50 patients who have undergone a brain surgery for either writer’s cramp or musician’s dystonia are reported in the literature; (2) all of these patients were operated in either Korea or Japan; (3) the majority were treated with ventralis oralis thalamotomy, the rest with thalamic DBS; and (4) the results were uniformly positive, though assessed in an un-blinded fashion with relatively short follow-up (178–181). There is virtually no literature regarding the use of pallidal surgery (ablation or DBS) for focal limb dystonia.

Given these reported results, the fact that DBS is a safe intervention in skilled hands (incidence of serious neurologic injury: 1–2%), and the opportunity to address an unmet need with this targeted intervention, it would seem that a more rigorous evaluation of thalamic DBS for focal limb dystonia is appropriate. However, the small but real risk of catastrophic stroke/hemorrhage and the fact that focal limb dystonia is neither life-threatening nor always debilitating, mandate that these studies be conducted at comprehensive movement disorders centers that include both an experienced DBS surgeon with a documented low surgical complication rate and neurologists facile both in the treatment of focal limb dystonia and the programming of DBS devices.

### Current Knowledge Gaps and Areas of Controversy in Therapy in Focal Limb Dystonia

Botulinum toxin therapy is often applied in an off-label manner in focal limb and task-specific dystonia but only limited evidence supports this practice due to the heterogeneity of the condition and to a lack of standardization in practice and data collection. Despite limitations, studies of rehabilitation in limb dystonias, as well as anecdotal reports, suggest a potential for improved outcomes for patients with rehabilitation intervention delivered by a therapist trained in the unique needs of a patient with dystonia, but definitive efficacy of a specific approach remains to be demonstrated. Non-invasive (rTMS and tDCS) and invasive (DBS) therapeutic modalities have been explored in only a small number of limb and task-specific dystonia patients and in studies with design limitations, which hampers the ability to move forward currently to larger clinical trials and to expand these potential therapies into clinical practice.

### Key Research Priorities in Therapy in Focal Limb Dystonia

- BoNT: refine the role of BoNT therapy by optimizing practice, developing new formulations, and use of combination therapeutic modalities (such as BoNT combined with physical therapy or neuromodulation)
- Rehabilitation: determine appropriate controls, understand the neurophysiological effects of rehabilitation for limb dystonias, determine best frequency and duration for interventions given that a long period of time likely is required for symptom development, determine duration of benefits after rehabilitation interventions
- Non-invasive brain stimulation: future trials to take into consideration the dose and duration of stimulation protocol, predictive markers for responders, designs which allow between and within subject effects to be explored and combination with specific motor retraining procedures
- Invasive brain stimulation: design randomized controlled trials with good subject characterization
- Identifying the triggering movement, and differentiating primary vs. compensatory movements is critical for selection of the best muscles for BoNT injection in musician’s dystonia
- Development of an effective therapeutic strategy including early identification of patients, prompt initiation of treatment as well as new and better therapies, and modification of the “every three months” BoNT injection paradigm to fit the schedule and needs of a performing artist

## Summary

Focal limb dystonias, from the task-specific to the peripherally induced, share clinical features with the other focal dystonias such as the adult-onset nature of the disease and the presence of sensory tricks that can temporarily ameliorate dystonic symptoms. However, the focal limb dystonias have a clinical heterogeneity (e.g., pianists dystonia and RD), which makes design of studies complicated from choosing specific anatomical targets for therapeutic interventions to developing comprehensive outcome measures that can fully quantify change in symptoms given high variability at baseline (Table 2). Focus on the research priorities as outlined here aims both to advance diagnostic capabilities and knowledge of the pathophysiology of this disorder, but also, to develop innovative therapeutic strategies to keep focal limb dystonia patients writing, running and performing.

**Table 2:** **Themes in focal limb dystonia research priorities**.

Diagnosis	Development of diagnostic criteria	Upper limb Lower limb Peripherally induced
	Standardize neurophysiologic tests	CMA Paired associative stimulation (PAS)
	Development of diagnostic battery using neurophysiology and imaging tests	Somatosensory temporal discrimination threshold Functional magnetic resonance imaging

Phenotypic characterization	Isolated focal limb dystonia	Relationship to neurodegenerative disease
	Peripherally induced dystonia	Identify factors that are protective or promoting
	Tremor, dystonia, dystonic tremor	Clarify the relationship of tremor with dystonia
	Genetic and environmental influences	Isolated limb dystonia and task-specific dystonia

Pathophysiology	Loss of inhibition	Understand how a loss at a network level translates to a focal symptom
	Abnormal plasticity	Understand the variability in PAS response
	Task specificity	Understand the relationship between repetition and abnormal plasticity
	Peripherally induced, posttraumatic	Understand commonalities and differences between isolated dystonia and posttraumatic

Therapy	Clinical trial development	Innovative designs with small *n* Duration of therapy needed for a disease that took years (or decades) to develop Harness the inter-patient variability Standardize outcome measures
	Development of therapeutic targets for invasive and non-invasive neurostimulation	Target localization for all focal limb dystonias Systematic assessment of duration and stimulation parameters

## Author Notes

This summary is a synopsis of the presentations prepared for 7th Annual Dystonia Coalition Meeting focusing on research priorities. The meeting was supported by a U54 grant from the NIH provided by the Office of Rare Diseases Research of the National Center for Advancing Translational Sciences (TR001456) in collaboration with the National Institute for Neurological Disorders and Stroke (NS065701). Administrative and material support was also provided by the Dystonia Medical Research Foundation. Travel grant support was provided by Beat Dystonia, the Benign Essential Blepharospasm Research Foundation, Cure Dystonia Now, the Dystonia Medical Research Foundation, and the National Spasmodic Dysphonia Association.

## Author Contributions

SPR, HJ, JP, RA, JJ, CL, RC, CP, TK, SFrucht, KA, EA, SFuruya, and MH made substantial contributions to the conception and design of the work; contributed to early drafts; revised critically for important intellectual content; approved the final version to be published; and, agreed to be accountable for all aspects of the work.

## Conflict of Interest Statement

EA is supported by the German Research Foundation (Al 279-2). KA is supported by the NIH Clinical Center Intramural Program. RA, SFrucht, SFuruya, and CL report no disclosures. RC is supported by the Canadian Institutes of Health Research, Catherine Manson Chair in Movement Disorders, Medtronic Inc., and Merz. He was a consultant for Merz, Allergan, and UCB. MH is supported by the NINDS Intramural Program. MH serves as Chair of the Medical Advisory Board for and may receive honoraria and funding for travel from the Neurotoxin Institute. He may accrue revenue on US Patent #6,780,413 B2 (Issued: August 24, 2004): Immunotoxin (MAB-Ricin) for the treatment of focal movement disorders, and US Patent #7,407,478 (Issued: August 5, 2008): Coil for Magnetic Stimulation and methods for using the same (H-coil); in relation to the latter, he has received license fee payments from the NIH (from Brainsway) for licensing of this patent. Supplemental research funds have been granted by UniQure for a clinical trial of AAV2-GDNF for Parkinson Disease, Merz for treatment studies of focal hand dystonia, and Allergan for studies of methods to inject botulinum toxins. JJ has received research and/or training grants from Allergan, Inc., Dystonia Medical Research Foundation, Medtronic Neuromodulation, Merz Pharmaceuticals, National Institutes of Health, Revance Therapeutics, Inc, St. Jude Medical, and has served as a consultant or as an advisory committee member for: Adamas Pharmaceuticals, Inc.; Allergan, Inc.; Pfizer; Teva Pharmaceutical Industries Ltd. HJ has active grant support from the US government (National Institutes of Health), private philanthropic organizations (the Benign Essential Blepharospasm Research Foundation, Cure Dystonia Now), and industry (Merz Inc., Ipsen Inc.). HJ also recently served as a consultant for Psyadon Pharmaceuticals, Saol Therapeutics, and Medtronic, Inc. HJ serves on the Scientific Advisory Boards for Cure Dystonia Now, the Dystonia Medical Research Foundation, Lesch-Nyhan Action France, the Lesch-Nyhan Syndrome Children’s Research Foundation and Tyler’s Hope for a Cure. He also is principle investigator for the Dystonia Coalition, which receives the majority of its support through NIH grant TR001456 from the Office of Rare Diseases Research at the National Center for Advancing Translational Sciences, and NS065701 from the National Institutes of Neurological Disorders and Stroke. The Dystonia Coalition has received additional material or administrative support from industry sponsors (Allergan Inc. and Merz Pharmaceuticals) as well as private foundations (The American Dystonia Society, Beat Dystonia, The Benign Essential Blepharospasm Foundation, Cure Dystonia Now, Dystonia Inc., Dystonia Ireland, The Dystonia Medical Research Foundation, The European Dystonia Federation, The Foundation for Dystonia Research, The National Spasmodic Dysphonia Association, and The National Spasmodic Torticollis Association). TK is supported in part by NIH R21DC012344 and R01DC015216. JP is supported by NIH/NINDS/NCATS/NIA (NS41509, NS075321, NS058714, NS092865, U10NS077384), the American Parkinson Disease Association (APDA), Greater St. Louis Chapter of the APDA, Barnes Jewish Hospital Foundation (Elliot Stein Family Fund, Oertli Fund), CHDI, and Huntington Disease Society of America. SPR is supported in part by NCRR/NCATS KL2 1 TR001448-01. CP is supported by the University of Minnesota’s MnDRIVE (Minnesota’s Discovery, Research and Innovation Economy) initiative.

## Abbreviations

BOLD, blood oxygen level dependent; BoNT, botulinum neurotoxin; CSP, cortical silent period; DBS, deep brain stimulation; DMN, default more network; fMRI, functional magnetic resonance imaging; GPi, globus pallidus interna; LTP, long-term potentiation; M1, motor cortex; PAS, paired associative stimulation; PET, positron emission tomography; PMC, premotor cortex; RD, runner’s dystonia; rTMS, repetitive transcranial magnetic stimulation; STDT, somatosensory temporal discrimination threshold; tDCS, transcranial direct current stimulation; TMS, transcranial magnetic stimulation.
